# Purification and Characterization of Native and Vaccine Candidate Mutant Enterotoxigenic *Escherichia coli* Heat-Stable Toxins

**DOI:** 10.3390/toxins10070274

**Published:** 2018-07-03

**Authors:** Morten L. Govasli, Yuleima Diaz, Ephrem Debebe Zegeye, Christine Darbakk, Arne M. Taxt, Pål Puntervoll

**Affiliations:** 1 Centre for Applied Biotechnology, Uni Research Environment, 5006 Bergen, Norway; Morten.Larsen@uni.no (M.L.G.); Yuleima.Diaz@uib.no (Y.D.); Ephrem.Zegeye@uni.no (E.D.Z.); 2 Department of Biological Sciences, University of Bergen, 5006 Bergen, Norway; Christine.Darbakk@student.uib.no (C.D.); Arne.Taxt@uib.no (A.M.T.); 3 Centre for International Health, University of Bergen, 5006 Bergen, Norway

**Keywords:** Enterotoxigenic *Escherichia coli*, ETEC, diarrhea, enterotoxin, heat-stable toxin, ST, disulfide isomerase, DsbC

## Abstract

Enterotoxigenic *Escherichia coli* (ETEC), which secretes the heat-stable toxin (ST) is among the four most important enteropathogens that cause moderate-to-severe diarrhea in children in low- and middle-income countries. ST is an intestinal molecular antagonist causing diarrhea and hence an attractive vaccine target. A non-toxic and safe ST vaccine should include one or more detoxifying mutations, and rigorous characterization of such mutants requires structurally intact peptides. To this end, we established a system for purification of ST and ST mutants by fusing the sequence encoding the mature ST peptide to the disulfide isomerase DsbC. A Tobacco Etch Virus protease cleavage site facilitates the proteolytic release of free ST with no additional residues. The purified ST peptides have the expected molecular masses, the correct number of disulfide bridges, and have biological activities and antigenic properties comparable to ST isolated from ETEC. We also show that free DsbC can assist in refolding denatured and misfolded ST in vitro. Finally, we demonstrate that the purification system can be used to produce ST mutants with an intact neutralizing epitope, that two single mutations, L9S and A14T, reduce toxicity more than 100-fold, and that the L9S/A14T double mutant has no measurable residual toxicity.

## 1. Introduction

Enterotoxigenic *Escherichia coli* (ETEC) is a common cause of diarrhea, and is an important cause of death as well as of malnutrition in children under the age of five in low- and middle-income countries (LMICs) [1,2,3]. ETEC is transmitted by the fecal-oral route and colonizes the small intestine [4]. Attachment to the intestinal epithelium is mediated by colonization factors, which are filamentous protein structures on the bacterial surface [5]. ETEC that infect humans are defined by secretion of at least one of three enterotoxins: the heat-labile toxin (LT) or the heat-stable toxins (ST), of which two variants exist, human (STh) and porcine (STp) [4,6]. The STs are secretory diarrhea-inducing toxins. ST-secreting ETEC (either ST-only or ST/LT), which comprise most ETEC strains, seem to cause the most serious cases of dehydrating ETEC diarrhea [7,8,9,10,11]. Hence, ST is an attractive target to include in an ETEC vaccine [12].

STh and STp are produced as 72-amino acid precursors that are processed into active toxins of 19 and 18 amino acids, respectively, which are stabilized by three intramolecular disulfide bridges [13]. ST binds to and activates the guanylate cyclase-C receptor (GC-C) that is present on the surface of epithelial cells in the gut and causes a rise in intracellular cyclic guanosine monophosphate (cGMP). Elevated levels of cGMP lead to downstream activation of the GC-C signaling pathways, which results in a net efflux of water and ions into the gut lumen [14]. This presents clinically as diarrhea.

The principal challenges to ST vaccine development are to render ST immunogenic, to detoxify the molecule without disrupting or masking important epitopes [4], and to avoid immunological cross-reactivity with the human peptides guanylin and uroguanylin [15,16]. ST has been made immunogenic by coupling it to a carrier molecule, either by genetic fusion [17,18,19,20,21,22,23], chemical conjugation [24,25,26], or by ligation of ST to a module consisting of a T-helper cell epitope and a Toll-like receptor 2 agonist [27]. Toxicity can be reduced or abolished by coupling ST to a carrier [17,28,29], but a safe ST immunogen should also include a detoxifying mutation [4,30]. We have screened a library of 361 single amino acid mutants of STh for effects on toxicity and the ability to bind to neutralizing antibodies, which led to the identification of mutants with low or no toxicity but intact epitopes [16]. Mutation may also be needed to minimize the risk of immunological cross-reactivity [15,16]. The results from the mutational screen can be used to guide the rational design of a safe and protective ST vaccine. However, to perform detailed characterizations of specific mutants, including their toxic and antigenic properties, structurally intact ST mutant peptides with no interfering additional amino acids are required.

ST peptides are typically isolated from ETEC supernatants and purified by labor-intensive protocols, involving adsorption, size-exclusion, and reversed-phase chromatography [31,32,33]. However, the process is challenging, might require time-consuming modifications for some mutants, and the protocols are apparently incompatible with the purification of some ST mutants [34]. In an alternative approach, ST was expressed as a fusion to the prosequence of uroguanylin in the cytoplasm of *E. coli*. To promote disulfide bridge formation, this approach used the *E. coli* Origami B (DE3) strain, which has an oxidative cytoplasm. By further using a protocol requiring two enzymatic cleavage steps, biologically active ST peptide from the fusion protein was obtained [35].

In a high throughput screen to identify efficient recombinant expression conditions for disulfide-rich proteins, the disulfide isomerase DsbC was identified as an attractive fusion partner for cytoplasmic expression [36]. Interestingly, the *E. coli* BL21 (DE3) pLysS strain with reducing cytoplasm was in general more efficient than strains with oxidative cytoplasm in producing soluble and functional disulfide-rich proteins. In the current study, we present an ST purification protocol that uses DsbC as a fusion partner to promote disulfide bridge formation, investigate the refolding activity of free DsbC on denatured and misfolded STh in vitro, and characterize a panel of vaccine candidate STh mutants.

## 2. Results

### 2.1. Expression and Purification of STh

**Figure 1:**
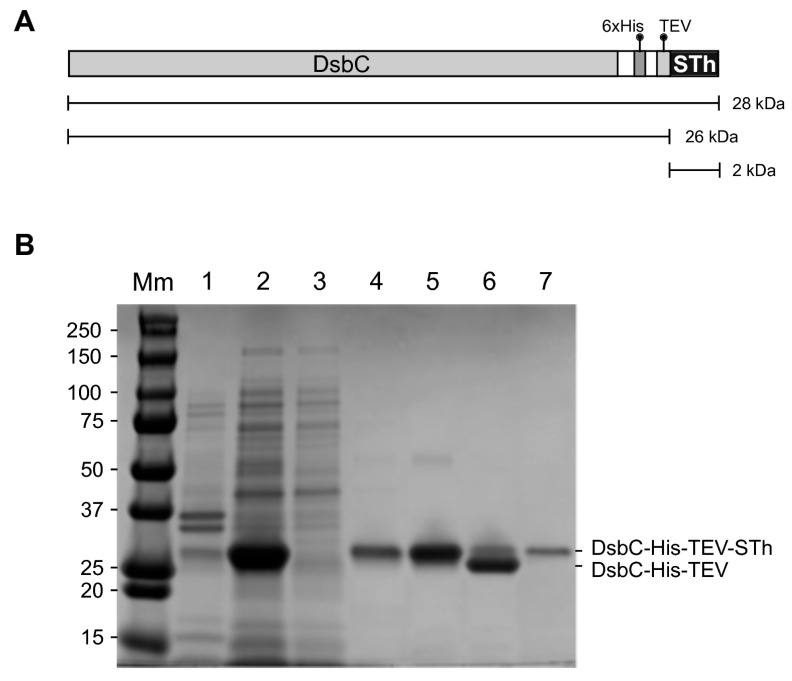
Purification of the DsbC-His-TEV-STh fusion protein and release of free STh peptide. (**A**) Cartoon of the DsbC-His-TEV-STh fusion protein with molecular mass of the full-sized fusion protein and those of the products after cleavage by the TEV protease: DsbC-His-TEV and STh. (**B**) SDS-PAGE gel showing expression of the DsbC-His-TEV-STh fusion protein in *E. coli* and the different steps of the purification protocol. Molecular masses (kDa) of the protein standard are shown to the left. Lane Mm: Molecular marker. Lane 1: resuspended pellet after sonication, centrifugation to remove cell debris, and removal of the clarified lysate. Lane 2: clarified lysate after sonication and centrifugation to remove debris. Lane 3: Ni-NTA agarose column flowthrough. Lane 4: wash. Lane 5: eluate from the Ni-NTA agarose column after buffer exchange. Lane 6: sample after overnight cleavage with TEV protease. Lane 7: TEV protease; note that it migrates with the same apparent mass as the full-length fusion protein. Both the fusion protein and TEV are removed in a second Ni-NTA agarose purification step (not shown). The STh peptide was collected in the flowthrough. The 2 kDa STh peptide is not visible on the gel. The location of the full-length and cleaved fusion proteins is indicated to the right.

To establish a simple purification system for the ST peptides, we genetically fused the sequence encoding the mature STh peptide immediately downstream of a sequence encoding DsbC, a 6xHis-tag, and a Tobacco Etch Virus (TEV) protease cleavage site (Figure 1A). The DsbC-His-TEV-STh fusion protein was expressed in *E. coli* BL21 Star™ (DE3), and the purification from the clarified lysate was monitored by SDS-PAGE (Figure 1B).

A dominant band with the expected molecular mass was observed in the clarified lysate, and very little fusion protein appeared to be present in the resuspended pellet, suggesting both good expression and solubility. The fusion protein was efficiently captured using a column packed with Ni-charged affinity resin (Ni-NTA agarose), but some protein was lost in the wash. The eluted fusion protein had a high purity, and the subsequent cleavage with the TEV protease led to a shift in mobility corresponding to the loss of STh. Due to its small size, STh is not visible on the gel. It is difficult to accurately estimate the cleavage efficiency from the SDS-PAGE as the TEV protease has a similar molecular mass as the full-length fusion protein.

**Figure 2:**
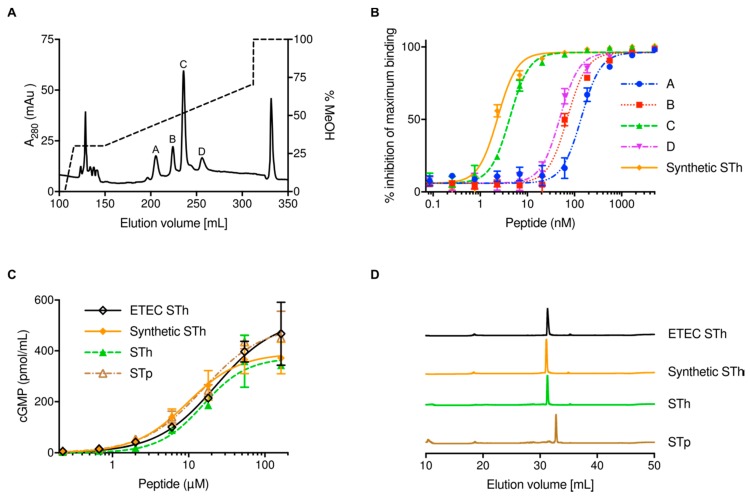
Purification and characterization of the STh peptide. (**A**) Chromatogram showing the elution of STh and isomers during reverse-phase high-performance liquid chromatography (RP-HPLC). STh-containing the flowthrough from Ni-NTA agarose removal of the fusion partner was applied to a preparative C18 reverse phase column. A linear gradient of methanol from 20–70% was used to elute peptides in RP-HPLC. The elution profile is shown as a solid line (left vertical axis), and the methanol gradient is shown as a dashed line (right vertical axis). The four main peaks are labelled. (**B**) Competitive ELISA to estimate the ability of the peptides from the RP-HPLC peaks to bind to the anti-STp C30 monoclonal antibody. The analysis was performed with serial dilutions of all peptides (horizontal axis; logarithmic scale). The symbols represent means of three independent assays, and error bars (standard deviations) are shown where they exceed the size of the symbol. The vertical axis represents the ability of the peptides to inhibit the binding of the antibody to the ELISA coating, given as a percentage of maximum binding measured in the absence of a competing peptide. Calculated half-maximal inhibitory concentrations (IC_50_s) are 139 ± 8 nM for peak A, 71 ± 4 nM for peak B, 4.2 ± 0.3 nM for peak C, 49 ± 3 nM for peak D, and 2.3 ± 0.1 nM for the control synthetic STh peptide. (**C**) T84 cell assay comparing the biological activity of the purified STh peptide (from peak C) to purified STp using the DsbC-ST system, synthetic STh, and STh purified from culture supernatants of ETEC. The analysis was performed with serial dilutions of all peptides (horizontal axis; logarithmic scale). The symbols represent mean values of three independent experiments, and error bars (standard deviations) are shown where they exceed the size of the symbol. (**D**) Analytical RP-HPLC elution profiles (A_280_) of the two DsbC-ST system peptides (STh and STp) compared to synthetic STh and STh purified from ETEC.

The STh peptide-containing flowthrough from the second Ni-NTA agarose purification was loaded onto a C18 column, a methanol gradient from 20–70% was used to elute bound STh, and four peaks were observed (Figure 2A).

Fractions from each peak were pooled, concentrated, and analyzed by mass spectrometry. All fractions contained one peptide with the same observed mass, 2041.54 Da, which indicates that they all contain isomers of STh with three disulfide bridges; STh with intact disulfide bridges has a theoretical monoisotopic mass of 2040.66 Da. Next, the STh peptides from the four peaks were analyzed by a competitive ELISA using the C30 anti-STp monoclonal antibody (Figure 2B). Peak C contained STh with an antigenicity almost identical to that of the synthetic STh control peptide; the difference in their half-maximal inhibitory concentration (IC_50_) values was not significant (*p* = 0.97). In contrast, the other peaks had significantly reduced IC_50_ values (*p* < 0.0001) compared to the control: peak A had 58-fold lower IC_50_, peak B had 30-fold lower IC_50_, and peak D had 20-fold lower IC_50_. This strongly suggests that only peak C contains correctly folded STh with an intact C30 epitope, which was hence used as the final purified product. Interestingly, only one peak was observed in the final C18 chromatography step when STp was purified with the DsbC system (data not shown). The biological activity of purified STh and STp peptides using the DsbC-ST system was compared to the activity of synthetic STh and STh purified from the supernatant of ETEC (ETEC STh) in the T84 cell assay (Figure 2C). The biological activities of the four peptides were very similar, as reflected by the calculated half-maximal effective concentrations (EC_50_s): 15.4 ± 3.0 μM for STh, 15.4 ± 3.8 μM for STp, 9.6 ± 1.8 μM for synthetic STh, and 22.9 ± 6.8 μM for ETEC STh. This suggests that the ST peptides purified with the DsbC-ST system had full biological activity. RP-HPLC of the four ST peptides revealed that the three STh peptides had comparable elution profiles, which were distinct from that of STp (Figure 2D). Moreover, the RP-HPLC profiles of STh and STp purified with the DsbC-ST system suggested that they contain one isomer and have high purity. Typical ST peptide yields from 1 L culture using the DsbC-ST system was 0.5–1 mg for STh, and 0.1–0.2 mg for STp.

### 2.2. The Disulfide Isomerase DsbC Can Refold Misfolded STh In Vitro

**Figure 3:**
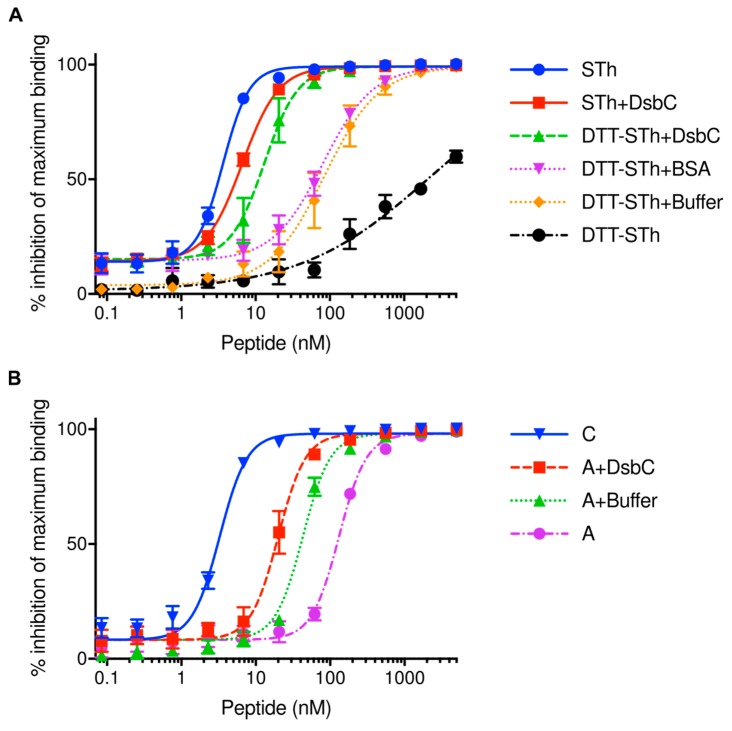
DsbC assisted refolding of STh. (**A**) STh was denatured with DTT and heat and subjected to different refolding conditions. The effect of refolding was monitored using the competitive C30 mAb ELISA. Three independent ELISAs were performed. Explanation of legends with calculated IC_50_s (nM): STh, STh peptide purified using the DsbC-His-TEV system (3.6 ± 0.2); DTT-STh, denatured STh (2111 ± 250); DTT-STh+Buffer, DTT-STh incubated in GSH/GSSG buffer containing reduced and oxidized glutathione (85 ± 5); DTT-STh+BSA, DTT-STh incubated with BSA in GSH/GSSG buffer (81 ± 6); DTT-STh-DsbC, DTT-STh incubated with DsbC in GSH/GSSG buffer (13 ± 1); STh+DsbC, STh incubated with DsbC in GSH/GSSG buffer (6.6 ± 0.4). (**B**) Refolding of the STh peptide from peak A compared to correctly folded STh (peak C; Figure 2A) monitored using the competitive C30 mAb ELISA. Three independent ELISAs were performed. Explanation of legends with calculated IC_50_s (nM): A, STh peptide from peak A (132 ± 6); A+Buffer, A incubated in GSH/GSSG buffer containing reduced and oxidized glutathione (43 ± 2); A+DsbC, A incubated with DsbC in GSH/GSSG buffer (20 ± 1); C, STh peptide from peak C (3.26 ± 0.2). The analysis in figure (**A**,**B**) was performed with serial dilutions of the peptides (horizontal axis; logarithmic scale). The symbols represent mean values of three independent experiments, and the error bars depict standard deviations.

To assess the DsbC activity directly, we denatured the STh peptide and used DsbC to refold it. STh was denatured using 10 mM DTT at 56 °C for 1 h, followed by buffer exchange to remove DTT. This resulted in DTT-STh with an IC_50_ value of 2111 ± 250 nM in the ST ELISA using the C30 mAb, which is more than 500-fold lower than that of native STh (3.6 ± 0.2 nM, Figure 3A).

All refolding reactions were performed in a buffer containing reduced and oxidized glutathione (GSH/GSSG) for 1h at room temperature. The refolding buffer had an intrinsic refolding effect on the DTT-STh, as demonstrated by the change in IC_50_ value to 85 ± 5 nM (DTT-STh+Buffer, Figure 3A). By adding DsbC, the IC_50_ value decreased even further to 13 ± 5 nM, suggesting that DsbC actively contributes to ST refolding. Note that the IC_50_ value of the control sample with protein bovine serum albumin (BSA) was not significantly different to that of buffer alone (*p* > 0.9999), suggesting no refolding effect. DsbC was also added to native STh as a control, resulting in a small but insignificant reduction in IC_50_ (*p* > 0.9999). Although the DsbC-assisted refolded DTT-STh peptide had a 3.6-fold lower IC_50_ than native STh, the difference was not significant (*p* = 0.999).

Three of the four STh-containing peaks observed in the RP-HPLC purification step appeared to contain STh isomers that were apparently incorrectly folded (Figure 2A,B). To assess whether it is possible to refold such isomers, we subjected the STh isomer from peak A, which had the highest IC_50_ value, to GSH/GSSH buffer and DsbC-assisted refolding (Figure 3B). Buffer alone decreased the IC_50_ value of the peak A in the C30 competitive ELISA significantly (*p* < 0.0001), and by adding DsbC the IC_50_ value was decreased further, significantly different from the buffer alone refolded peak A STh (*p* < 0.0001). However, the DsbC-assisted refolded peak A STh had an IC_50_ that was 6.1-fold higher than that of the native peak C STh, but 6.6-fold lower than that of peak A isomer (*p* < 0.0001). In conclusion, DsbC in GSH/GSSG buffer efficiently refolded both denatured and misfolded STh.

### 2.3. Characterization of Vaccine Candidate STh Mutants

**Figure 4:**
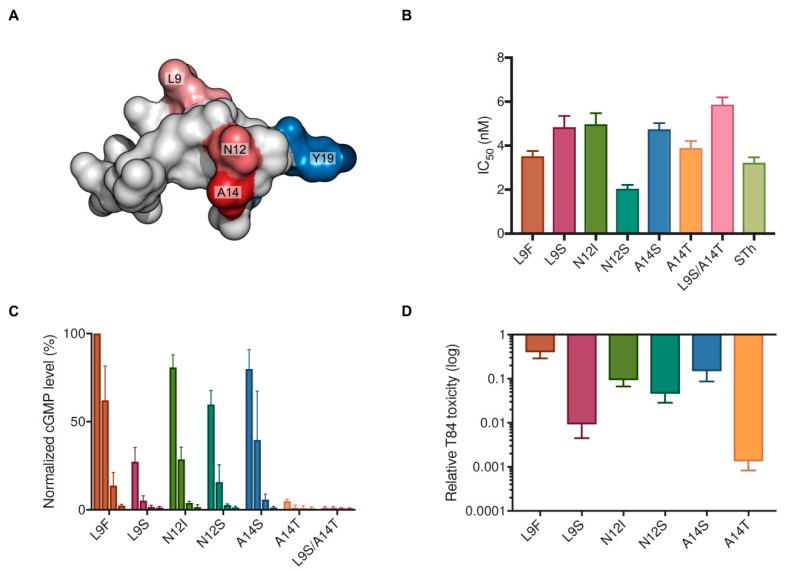
Characterization of purified STh mutants. (**A**) Structural model of STh highlighting residues that were targeted for mutation in red (L9, N12, A14) and Y19 in blue, which is the main epitope residue of the C30 epitope [16]. (**B**) The effect of binding of the STh mutant peptides to the C30 mAb was assessed using competitive ELISA. Three independent experiments were performed, and the mean calculated IC_50_ values with standard deviations are shown for each mutant. (**C**) Dose-response analysis of the mutant STh peptides in the T84 cell assay. Four concentrations were used for each peptide (shown from left to right): 1627 nM, 163 nM, 16 nM, and 1,6 nM. Three independent experiments were performed, and the mean normalized cGMP response with standard deviations is shown for each mutant. The cGMP levels were normalized relative to native STh (100%) (**D**) The toxicities as observed in the T84 cell assay of the single mutant STh peptides relative to that of native STh. The relative toxicities were calculated from a standard curve of native STh, and the error bars depict the standard deviations from three individual experiments. L9S/A14T is not shown as it had no measurable residual toxicity.

To test the suitability of the DsbC-ST system for STh mutant production, a small panel of mutants were selected for purification and characterization. ST mutants suitable for a vaccine should have no or low toxicity, but intact epitopes. In a screen of all 361 single amino acid mutants of STh, the top 30 vaccine candidate mutants all had mutations in residues L9, N12, and A14 (Figure 4A) [16]. We picked two mutants from each position: one predicted to have a modest effect on toxicity (L9F, N12I, A14S), and one to have a more pronounced effect on toxicity (L9S, N12S, A14T). In addition, we constructed a double mutant L9S/A14T by combining the predicted low toxicity mutants of residues L9S and A14T.

All mutants were successfully purified as revealed by their RP-HPLC elution profiles (Figure S1), and had the expected molecular masses as confirmed by mass spectrometry. Furthermore, the mutant peptides had comparable affinities to the C30 mAb (Figure 4B), as reflected by similar IC_50_ values in the competitive ELISA, ranging from 2.0–5.9 nM (native STh, IC_50_ = 3.2 nM). This suggests that the C30 epitope was intact in all mutants, and that the introduced mutations did not disrupt the peptide structure. The effect of mutations on toxicity was tested in the T84 cell assay using four different concentrations of each peptide: 1627 nM, 163 nM, 16 nM, and 1.6 nM (Figure 4C). All peptides displayed detectable toxicities, except for the L9S/A14T double mutant (Figure 4C), and all mutants had reduced toxicities compared to native STh (Figure 4D). As predicted, the L9F, A14S, and N12I mutants had the lowest reductions in toxicity, with 3-fold, 8-fold, and 11-fold reductions, respectively. The mutants predicted to have more pronounced reductions in toxicities were N12S, L9S, and A14T, with 24-fold, 128-fold, and 820-fold reductions, respectively.

## 3. Discussion

ETEC strains secreting ST, with or without LT, are among the most important enteropathogens causing moderate to severe diarrhea in children under 5 years of age in LMIC [1,2,3]. Accordingly, ST is a prime target for vaccine development that may complement ongoing ETEC vaccine development efforts. In order to facilitate ST-based vaccine development, a versatile method for ST purification that ensures proper folding of the disulfide-rich ST peptide would be highly desirable. Here, we have developed a versatile recombinant ST purification method that utilizes the disulfide bond reshuffling activity of DsbC protein to promote native disulfide bridges in ST. We demonstrate that our system allows purification of the native toxin, as well as single, and double mutant of ST, including novel vaccine candidates. The system provides ST peptides of high purity and with sufficient yields for detailed characterization of toxicity and antigenicity. We further demonstrate that the fusion partner DsbC indeed promotes refolding of denatured and incorrectly folded ST.

We chose DsbC as a fusion partner for ST due to its reported ability to assist in correct disulfide bridge formation in cysteine-rich proteins and peptides during recombinant expression in the cytoplasm of *E. coli* [36]. The fact that we were able to purify both STh and STp with full biological activity using DsbC as a fusion partner suggests that DsbC exerts its disulfide isomerase activity also on the ST peptides. This was corroborated by the apparent ability of DsbC to assist in refolding of denatured and misfolded STh (Figure 3). Interestingly, we observed four major and distinct peaks in the preparative RP-HPLC (Figure 2A), all containing fully oxidized STh peptides with the same molecular mass. This suggests that STh can form at least four distinct disulfide bridge configurations. The three isomers with apparent non-native disulfide bridge configurations were bound by the C30 antibody with a significantly lower affinity, suggesting that the Y19-based epitope (Figure 4A), and by extension the peptide, had non-native structures. In the DsbC-ST system, the DsbC protein may exert its isomerase and chaperonin activity both in vivo and ex vivo [36]. The TEV cleavage reaction, which releases free ST peptides from the DsbC-His-TEV-ST fusion protein, was performed overnight in the GSH/GSSG buffer, giving ample time for DsbC to act on ST both when fused and as free peptides. Still, three apparently misfolded isomers were formed in addition to the correctly folded one. The fact that it was possible to refold one of the misfolded isomers by using the same buffer that was used during proteolytic cleavage and the DsbC protein suggests that the isomers form a dynamic equilibrium. Hence, it is conceivable that the purification system can be optimized by pushing this equilibrium towards correctly folded ST peptides.

Mutations are required to address two of the ST vaccine development challenges, namely to reduce or abolish toxicity, and to avoid eliciting antibodies that can cross-react with the human peptides guanylin and uroguanylin. The STh residues L9, N12, and A14 are all important for toxicity [4,16], and they are shared with uroguanylin (only A14 is shared with guanylin). This implies that both challenges may be met by mutating one or more of these residues. The screen of all 361 single amino acid mutants of STh for effects on toxicity and their ability to bind to neutralizing antibodies allowed us to rank mutants according to their antigenicity-toxicity ratio, which is a measure for their suitability as vaccine candidates [16]. The aim was to identify mutants with low or no toxicity but intact epitopes. The choice of mutants for each of the three residues was informed by the ranked list, but we also imposed an additional criterion to the selection. To minimize the risk of introducing neoepitopes that may eclipse native, neutralizing epitopes, we opted for the small polar amino acids serine and threonine. L9S ranked as the second-best L9 mutation, N12S as the sixth N12 mutation, and A14T as the fourth best A14 and overall mutation. As predicted, the A14T mutation had the most dramatic effect on toxicity, with an 820-fold reduction compared to native STh. This is in stark contrast to the A14S mutation, which differs from A14T by a single methyl group and only reduced the toxicity 8-fold. The N12S mutation is used in an ST vaccine candidate where three copies of STh-N12S is genetically fused to a non-toxic double mutant of the heat-labile toxin from ETEC [21,37]. Although the reduction in toxicity for the N12S mutation was modest (24-fold compared to native STh), the genetic fusion is reported to be non-toxic [21].

Our preferred strategy for making ST immunogenic is to couple ST (mutants) to a protein carrier by chemical conjugation. This approach allows full characterization of the structural integrity of mutants prior to conjugation, and the ability to obtain a high hapten-to-carrier ratio, which may enhance immunogenicity. We consider the single mutant A14T as a good candidate for inclusion in an ST conjugate vaccine. But to reduce the risk of unwanted immunological cross-reaction, a double mutant like the non-toxic L9S/A14T may be necessary. The results presented here demonstrate that the DsbC-ST system can be used to purify ST vaccine candidate mutants, and that the yield is sufficient for preparing ST-conjugates for testing in animals. However, this system is unlikely to be suitable for large-scale vaccine production, for which we anticipate that chemical synthesis will be more suitable. Interestingly, the successful DsbC-assisted refolding of ST suggests that it should be possible to use DsbC also to assist in the folding of chemically synthesized ST.

## 4. Materials and Methods 

### 4.1. Construction of Native and Mutant ST Expression Vectors

The vector pETDsbCin_1b was a generous gift from Gunter Stier [38]. This vector encodes the *E. coli* thiol:disulfide interchange protein DsbC fused to the yellow fluorescence protein (EYFP), linked by a 6x-histidine tag and a TEV protease site. The signal sequence of DsbC has been removed to allow cytoplasmic expression. The DNA sequences encoding mature STh and STp peptides, and STh mutants were introduced into the plasmid using appropriate primers (Table 1) and the Q5^®^ Site-Directed Mutagenesis Kit (New England Biolabs, Ipswich, UK). Rare *E. coli* codons were avoided in the primer design.

The primers introduce the ST sequences immediately following the TEV recognition site ENLYFQ and places the first STh and STp peptide residue N1 in the TEV cleavage site P1’ position [39]. Hence, the ST expression vectors (Table 2) were designed to permit the cleavage of ST peptides from the fusion partner with no additional N-terminal amino acids. Additionally, the primers introduced stop codons immediately after the ST sequences, preventing translation of the downstream EYFP sequence present in the vector backbone. All vectors were verified by sequencing.

To allow for the purification of DsbC alone, a stop codon was introduced after the DsbC coding sequence in the pETDsbCin_1b plasmid using the Q5^®^ Site-Directed Mutagenesis Kit and primers DsbC-TAA-F and DsbC-TAA-R (Table 1). This stop codon prevents EYFP from being included in the translation. The resulting plasmid, pET-DsbC (Table 2), was verified by sequencing.

### 4.2. Expression and Purification of DsbC-ST Fusion Proteins

The *E. coli* BL21 Star™ (DE3) strain (Invitrogen, Waltham, UK) was used for protein expression. In brief, the plasmids encoding DsbC-ST fusions (Table 2) were transformed into BL21 Star™ (DE3) following the manufacturer’s instructions. Transformed cells were plated onto Lysogeny broth (LB) agar plates supplemented with 50 µg mL^−1^ kanamycin. Single colonies were used to inoculate overnight starter cultures of 5 mL LB containing 50 µg mL^−1^ kanamycin, and cultures were grown at 37 °C in an orbital shaker at 250 rpm. The overnight starter cultures were then used to inoculate 1 L 2 × YT medium supplemented with 2% (*w*/*v*) glucose and 50 µg mL^−1^ kanamycin. The cultures were grown using orbital shakers at 37 °C and 250 rpm until the OD_600nm_ reached 0.6, and expression was induced by the addition of IPTG to a final concentration of 500 µM, the temperature was subsequently lowered to 18 °C, and incubated overnight.

After expression, cells were harvested by centrifugation at 6000× *g* for 20 min followed by resuspension in 10 mL lysis buffer (50 mM Tris-HCl, 300 mM NaCl, 20 mM Imidazole, 0.2% Triton X-100 [pH 8]) per gram wet weight cells. Lysozyme from chicken egg white (Sigma-Aldrich, St. Louis, MO, USA) was added to a concentration of 1 mg mL^−1^ to facilitate lysis. Subsequently, resuspended cells were lysed using an Ultrasonic homogenizer (Cole-Parmer Instrument Co., Vernon Hills, IL, USA) on ice, using 30-s intervals until complete lysis was achieved. Cell debris was removed by centrifugation at 20,000× *g* for 20 min. The cleared lysate was purified with Ni-NTA using a HisTrap FF Crude 5 mL column (GE healthcare life sciences, Chicago, IL, USA) connected to an ÄKTA pure system (GE healthcare life sciences). A flow rate of 3 mL min^−1^ was used for sample loading while a flow of 5 mL min^−1^ was used for equilibration, washing and elution. Purification was performed using buffer A (50 mM Tris-HCl, 300 mM NaCl, 20 mM Imidazole [pH 8]) and buffer B (50 mM Tris-HCl, 300 mM NaCl, 500 mM Imidazole [pH 8]).

### 4.3. Cleavage of DsbC-ST Fusion Proteins and Purification of ST Peptides

Following Ni-NTA agarose purification, the buffer was exchanged into TEV cleavage buffer (20 mM sodium phosphate, 125 mM NaCl [pH 7.4]) using PD-10 columns (GE healthcare life sciences) and the gravity protocol according to the manufacturer’s instructions. The 6x-histidine-tagged TEV was produced as described [40], but purified without the use of a reducing agent. Following the buffer exchange, reduced and oxidized glutathione ((GSH and GSSG) (Sigma–Aldrich)) were added to final concentrations of 0.6 mM and 0.4 mM, respectively [41], to obtain a redox potential that should keep TEV active while minimizing shuffling of the disulfide bridges of ST. Cleavage was initiated by adding TEV to the fusion protein at a 1:30 molar ratio, and cleavage was performed at room temperature overnight. After cleavage, the solution was passed over a Ni-NTA agarose resin (Qiagen, Hilden, Germany) once more to remove TEV, the DsbC-fusion partner, and uncleaved DsbC-ST fusions. The ST peptide containing flowthrough was collected and loaded onto a pre-equilibrated XBridge Peptide BEH C18 OBD Prep Column, 130 Å, 10 µm, 10 mm × 250 mm (Waters, Milford, MA, USA), connected to an ÄKTA pure system (GE healthcare life sciences). The system was operated with a flow rate of 2 mL min^−1^ for equilibration, sample loading, wash and elution. For purification Buffer A (0.3% acetic acid in Milli-Q [pH 5.6]) and buffer B (0.3% acetic acid in 100% methanol [pH 5.6]) was used. The pH for both buffers was adjusted with 25% NH_4_OH. Fractions from the main peaks were pooled and concentrated using a vacuum centrifuge before further characterization.

### 4.4. Competitive ST ELISA

The antigenicity of the purified ST peptides was examined with a previously described competitive ELISA [15], but with some modifications. In brief, Nunc Amino Immobilizer microtiter plates (Thermo Fisher Scientific, Waltham, MA, UK) were coated overnight at 4 °C with 100 µL ELISA PBS (3.25 mM Na_2_HPO_4_, 9.6 mM NaH_2_PO_4_, 146 mM NaCl [pH 7.4]) containing recombinant STh, resulting in a final amount of 4 ng STh per well. The following day, excess protein binding sites were blocked by adding 180 µL PBS-T (3.25 mM Na_2_HPO_4_, 9.6 mM NaH_2_PO_4_, 146 mM NaCl, 0.05% (*v*/*v*) Tween-20, [pH 7.4]) containing 1% (*w*/*v*) ovalbumin for one hour at room temperature with gentle shaking. Following blocking, 60 µL of the samples to be analyzed were added in desired concentrations, and subsequently 60 µL of the C30 anti-STp monoclonal antibody clone M120530 (Fitzgerald, North Acton, MA, UK) was added to a final concentration of 1:16000, allowing competition for the binding to the coating or to the peptide in solution for 1–2 h using gentle shaking at room temperature. ELISA PBS-T was used in all dilutions. 100 µL ELISA PBS-T containing a secondary anti-mouse antibody (Product code: ab6729 (Abcam, Cambridge, UK)) linked to alkaline phosphate was then added to a final dilution of 1:4000. After an hour of incubation, the plates were developed by adding 100 µL of diethanolamine buffer (250 mM diethanolamine, 0.5 mM MgCl_2_ [pH 9.8]) containing 0.5 mg 4-Nitrophenyl phosphate disodium salt hexahydrate mL^−1^. The absorbance at 405 nm was measured when total activity (no competing peptide) wells reached an absorbance between 1 and 2 using a Hidex Sense microplate reader (Hidex, Turku, Finland). Synthetic STh was used as a control.

### 4.5. T84 Cell Assay

The T84 cell assay was performed as described previously [15]. Briefly, T84 cells (ATCC, Rockville, MD, USA) were seeded and grown to confluence on Nunc 24-well plates (Thermo Fisher Scientific) in Gibco™ Dulbecco's Modified Eagle Medium: Nutrient Mixture F-12 ((DMEM/F-12) (Thermo Fisher Scientific)), supplemented with 10% fetal bovine serum (Sigma-Aldrich) and 0.2% gentamicin (Lonza, Basel, Switzerland). Cells were washed thrice with 500 µL DMEM/-F-12, and pre-incubated with 200 µL DMEM/F-12 containing 1 mM 3-isobutyl-1-methylxanthine (Sigma–Aldrich) for 10 min at 37 °C. A volume of 200 µL of sample was added to each well and incubated for 60 min at 37 °C. Following incubation, the reaction medium was aspirated, and cells were lysed with 0.1 M HCl at 20 °C for 20 min. The lysates were centrifuged at 16,000× *g* for 10 min and supernatants were collected for analysis. Levels of cGMP were determined using a cGMP enzyme immunoassay kit (Enzo Life Sciences, Inc, Farmingdale, NY, USA) according to the manufacturer's instructions. STh purified from ETEC was used as a control.

### 4.6. Mass Spectrometry

The peptides were analyzed using an ULTRAFLEX II (Bruker Daltonics, Bremen, Germany) MALDI-TOF/TOF mass spectrometer in positive ion reflector mode. 0.4 µL of the sample was mixed with 0.4 µL alpha-cyano-4-hydroxycinnamic acid (Bruker Daltonics) matrix solution (10 mg mL^−1^ in 0.1% trifluoroacetic acid/acetonitrile) in a 1:1 ratio and spotted onto a MALDI plate. For some peptides, the samples were purified using a µ-C18 ZipTip (Millipore, Billerica, MA, USA) and directly spotted onto the MALDI plate with 0.6 µL alpha-cyano-4-hydroxycinnamic acid. The mass spectra were internally calculated from the raw mass spectra using the SNAP algorithm in FlexAnalysis 2.4 (Bruker Daltonics). Expected masses were calculated using the PeptideSynthetics Peptide mass calculator [42].

### 4.7. Analytical Reversed-phase High-performance Liquid Chromatography

The purity of all the ST peptides was assessed with analytic RP-HPLC using a Nucleodur EC C18 gravity, 3 µm, 250 × 3 mm (Macherey-Nagel, Düren, Germany) column operated with an Agilent 1200 Series system (Agilent Technologies, Santa Clara, CA, USA). Before analysis, the peptide samples were diluted to yield concentrations of 100 µM in PBS (137 mM NaCl, 2.7 mM KCl, 10 mM Na_2_HPO_4_, 1.8 mM KH_2_PO_4_ pH [7.2]). The HPLC column was equilibrated with buffer A (0.1% trifluoroacetic acid in Milli-Q) before the samples were injected. The STs were eluted with buffer B (0.1% trifluoroacetic acid in 100% acetonitrile) using a gradient from 15 to 65% buffer B over 30 min. The system was operated with a flow rate of 350 µL min^−1^ and the absorbance was recorded at 220 nm.

### 4.8. DsbC Assisted In Vitro Refolding of Denatured STh

DsbC was expressed and purified using Ni-NTA agarose as described above for the DsbC-STh fusion proteins. Following elution, the buffer was changed into TEV cleavage buffer using PD-10 columns (GE healthcare life sciences) using the gravity protocol.

Purified native STh was denatured using 10 mM DTT at 56 °C for 1 h. Buffer exchange into PBS pH 7.2 was performed immediately afterwards using Spinout GT-100 columns (G-Biosciences, St. Louis, MO, USA). The DTT-reduced STh, hereafter called DTT-STh, was aliquoted and stored at −20 °C until refolding assays were carried out.

Refolding activity of DsbC was assayed by incubating 4 nmol of DTT-STh in TEV cleavage buffer (20 mM sodium phosphate, 125 mM NaCl, 1 mM EDTA [pH 7.4]), supplemented with 0.6 mM GSH and 0.4 mM GSSG, in the presence or absence of 8 nmol of DsbC (150 µL reaction). Similarly, disulfide isomerase activity of DsbC was assayed on an STh isomer with apparently incorrect disulfide bridge connectivity. To account for spontaneous refolding, a reaction lacking DsbC but containing TEV cleavage buffer, supplemented with 0.6 mM GSH and 0.4 mM GSSG, was included in both assays. Reactions took place at room temperature for 1 h. Thereafter, the concentration was adjusted to 10 µM using PBS-T, three-fold serial dilutions were prepared, and samples were analyzed by competitive ELISA as described above.

### 4.9. Data and Statistical Analyses

GraphPad Prism 7 was used to prepare plots and to perform statistical analyses. Four-parameter logistic regression was performed on competitive ELISA results to estimate the half-maximal inhibitory concentrations (IC_50_) and on T84 cell assay results to estimate the half-maximal effective concentration (EC_50_). For ELISA analyses of the RP-HPLC C18 peaks and the refolding of peak A, all parameters except IC_50_ were set to be shared for all datasets. For ELISA analysis of refolding of denatured ST, the top parameter was set to be shared for all data sets, and for the mutant peptides, no constraints were set. For T84 cell assay analysis of the native peptides, the bottom parameter was set to zero. Ordinary one-way ANOVA with Tukey’s multiple comparisons test was used to evaluate whether IC_50_ values in ELISAs and EC_50_ values in T84 cell assays differed significantly.

For T84 cell assay analysis of ST mutants, toxicities relative to native STh were calculated by using a standard curve of native STh. The standard curve was constructed by using a two-fold serial dilution of STh ranging from 163 nM to 0.2 nM. Each mutant peptide was assayed at four different concentrations: 1627 nM, 163 nM, 16 nM, and 1.6 nM, and the concentration that produced a cGMP response that fell in the linear range of the standard curve was used to read off the native STh concentration that would produce the same cGMP response. This native ST concentration was divided by the mutant concentration to calculate the relative toxicity. Each of the three replicates was analyzed individually. Asymmetric sigmoidal interpolation was used for two of the standard curves, and sigmoidal interpolation was used for the third.

## 5. Patents

Pål Puntervoll and co-workers have submitted a patent application (application no. 61/766,958) for the A14T toxoid, which is intended for use in an ETEC vaccine.

## Figures and Tables

**Table 1:** List of primers used to generate expression vectors for native STh and STp, and mutant STh peptides. ST encoding sequences are shown in lower case, and vector sequences are in upper case. Stop codons are underlined.

Primer	Nucleotide Sequence
STh-F	tgtaatcctgcttgtaccgggtgctattaaGGCGCCATGGGCAAAGTG
STp-F	taatcctgcttgtgctggttgctattaaGGCGCCATGGGCAAAGTG
STh-N12I-F	tgtatccctgcttgtaccgggtgctattaaGGCGCCATGGGCAAAGTG
STh-N12S-F	tgtagccctgcttgtaccgggtgctattaaGGCGCCATGGGCAAAGTG
STh-A14S-F	tgtaatcctagctgtaccgggtgctattaaGGCGCCATGGGCAAAGTG
STh-A14T(a)-F	tgtaatcctacttgtaccgggtgctattaaGGCGCCATGGGCAAAGTG
STh-A14T(b)-F	tgtaatcctacatgtaccgggtgctattaaGGCGCCATGGGCAAAGTG
DsbC-TAA-F	taaGCCATGGGCAAAGTGAGC
STh-R	acacaattcacagcagtaattgctactattCTGAAAATAAAGATTCTCGCTACCCG
STp-R	caacacaattcacagcagtagaaggtattCTGAAAATAAAGATTCTCGCTACCCG
STh-L9A-R	acatgcttcacagcagtaattgctactattCTGAAAATAAAGATTCTCGCTACCCG
STh-L9F-R	acaaaattcacagcagtaattgctactattCTGAAAATAAAGATTCTCGCTACCCG
STh-L9N-R	acagttttcacagcagtaattgctactattCTGAAAATAAAGATTCTCGCTACCCG
STh-L9S-R	acaactttcacagcagtaattgctactattCTGAAAATAAAGATTCTCGCTACCCG
STh-N12S-R	acacagttcacagcagtaattgctactattCTGAAAATAAAGATTCTCGCTACCCG
DsbC-TAA-R	GCCCTGAAAATAAAGATTCTCGC

**Table 2:** Expression vectors for native STh and STp, and STh mutants.

Plasmid	Forward Primer	Reverse Primer	ST Variant
pET-DsbC-STh	STh-F	STh-R	STh
pET-DsbC-STp	STp-F	STp-R	STp
pET-DsbC-STh-L9A	STh-F	STh-L9A-R	STh-L9A
pET-DsbC-STh-L9F	STh-F	STh-L9F-R	STh-L9F
pET-DsbC-STh-L9N	STh-F	STh-L9N-R	STh-L9N
pET-DsbC-STh-L9S	STh-F	STh-L9S-R	STh-L9S
pET-DsbC-STh-N12S	STh-N12S-F	STh-N12S-R	STh-N12S
pET-DsbC-STh-N12I	STh-N12I-F	STh-R	STh-N12I
pET-DsbC-STh-A14S	STh-A14S-F	STh-R	STh-A14S
pET-DsbC-STh-A14T	STh-A14T(a)-F	STh-R	STh-A14T
pET-DsbC-STh-L9S/A14T	STh-A14T(b)-F	STh-L9S-R	STh-L9S/A14T

## Supplementary Materials

The following are available online at http://www.mdpi.com/2072-6651/10/7/274/s1, Figure S1: Analytical RP-HPLC elution profiles (A_220_) of the mutant STh peptides compared to native STh.
