# Supplementary material for: Purification and Characterization of Native and Vaccine Candidate Mutant Enterotoxigenic Escherichia coli Heat-Stable Toxins
Source: Toxins (Basel). 2018 Jul 3;10(7):274. doi: 10.3390/toxins10070274 (PMC6071264; doi:10.3390/toxins10070274)
Supplement: Supplementary file 1 [file toxins-10-00274-s001.pdf]

Supplementary Materials: Purification and Characterization of Native and Vaccine Candidate Mutant Enterotoxigenic *Escherichia coli* Heat-stable Toxins

Morten L. Govasli, Yuleima Diaz, Ephrem Debebe Zegeye, Christine Darbakk, Arne M. Taxt, Pål Puntervoll

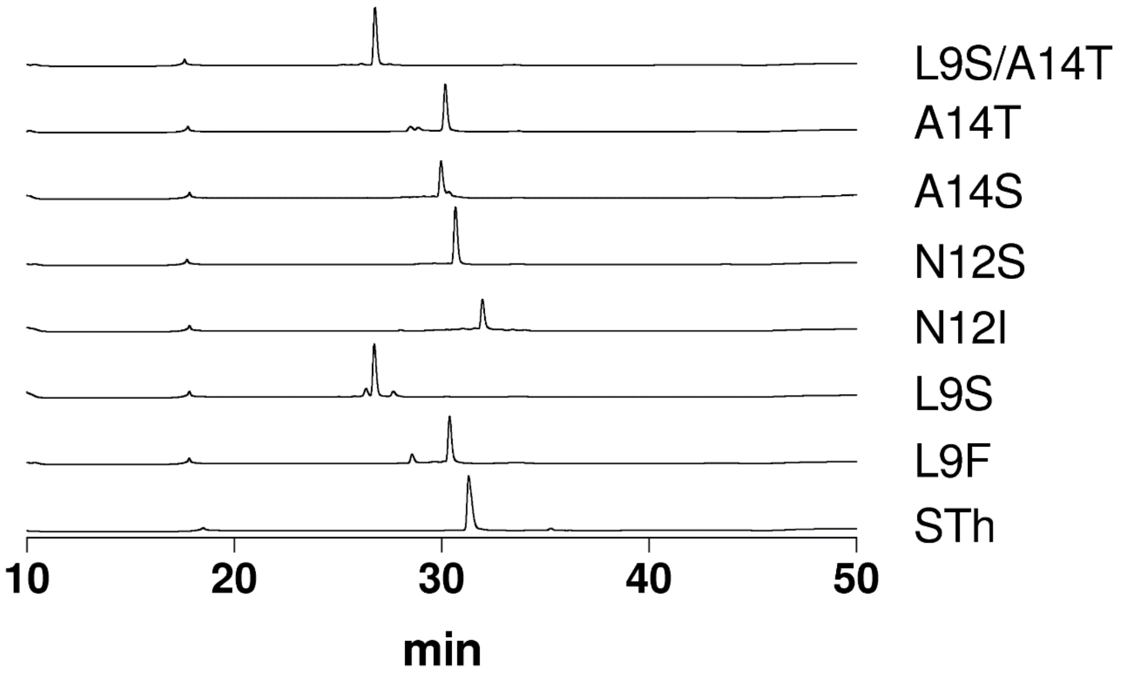

Figure S1. Analytical RP-HPLC elution profiles (A_{220}) of the mutant STh peptides compared to native STh.
